# Prevalence and Antimicrobial Susceptibility Patterns of *Neisseria gonorrhoeae* among Suspected Patients Attending Private Clinics in Jimma, Ethiopia

**DOI:** 10.1155/2020/7672024

**Published:** 2020-08-24

**Authors:** Addisu Sahile, Lule Teshager, Minale Fekadie, Mulatu Gashaw

**Affiliations:** ^1^School of Medical Laboratory Sciences, Jimma University, P.O. Box 378, Jimma, Ethiopia; ^2^School of Biomedical Sciences, Jimma University, P.O. Box 378, Jimma, Ethiopia

## Abstract

**Background:**

In most African countries including Ethiopia, *Neisseria gonorrhoeae* infections were diagnosed clinically and its antibiotic susceptibility was rarely tested. This study aimed to determine the prevalence and antimicrobial susceptibility patterns of *N. gonorrhoeae* among suspected patients attending private clinics in Jimma, Ethiopia.

**Methods:**

Institution-based cross-sectional study was conducted to determine the prevalence and antimicrobial susceptibility pattern of *N. gonorrhoeae* isolated from urogenital specimens. Urogenital samples were collected aseptically and then transported using Amie's transport media and processed in a microbiology laboratory following the standard protocol.

**Results:**

Of the total 315 samples examined, 31 (9.8%) were confirmed to have gonococcal infection. Of these, 30 (96.7%) were females. High proportion of culture confirmed cases (18 (12.5%)) were observed in the 20–24 age group. All of the identified organisms were susceptible to ceftriaxone and had high resistance to penicillin (80.6%) and tetracycline (54.8%).

**Conclusion:**

The prevalence of gonococcal infection is high. In the current study, participants who have no information about sexually transmitted infection were more likely to be infected by *N. gonorrhoeae.* According to our study, ciprofloxacin is effective against gonococcal infection.

## 1. Introduction

Gonorrhea is a sexually transmitted disease caused by *N. gonorrhoeae* for which humans are the only natural host. *N. gonorrhoeae* is one of the most common sexually transmitted diseases in developing countries [1]. It causes infections principally of the urethra in men and the endocervix in women, where the columnar epithelium of the endocervix is susceptible to infection and having any sign and symptom such as pain during sexual intercourse, a painful or burning sensation when urinating, and abnormal vaginal discharge, and, during disseminated infection, it causes cramps and pain to the lower abdomen [2]. In addition to causing serious complications, gonorrhea is a potent amplifier of the spread of sexually transmitted infections like HIV by increasing the entry of infective inoculums of the virus in persons coinfected with HIV and gonorrhea [2, 3].

Currently, in many studies, a high proportion of drug resistance was reported for *N. gonorrhoeae* [4]. Although extended-spectrum cephalosporin is the only first-line antimicrobials recommended for the empirical treatment of uncomplicated gonorrhea in many countries, many strains of *N. gonorrhoeae* with reduced susceptibility to cephalosporins have been reported worldwide [5]. Extensive drug-resistant *N. gonorrhoeae* strains have also been reported in Japan [6], France [7], and Spain [8] that displayed high-level resistance to cefixime and ceftriaxone. Resistance to commonly prescribed antibiotics is an expanding global problem resulting in diminishing treatment options for gonorrhea [9].

Loss of utility of several drugs such as sulfonamides, penicillin, and tetracycline, for treatment of gonorrhea, was reported in both developed and developing countries [10]. With the occurrence of resistance to commonly prescribed antibiotics in both developed and developing countries, the up-to-date treatment guidelines in African countries suggest the syndromic case management for all sexually transmitted infections (STIs) including gonorrhoeae based on the WHO recommendation which has high contribution to drug resistance by the bacteria [11].

Updated knowledge about the susceptibility patterns of gonococci in Ethiopia is important for the proper selection and use of antimicrobial as well as for the development of an appropriate prescription policy. The aim of this is to investigate the prevalence and antibiotic susceptibility patterns of *N. gonorrhoeae* isolated from urogenital samples from suspected patients attended in Jimma Town Private Clinics, Southwest, Ethiopia.

## 2. Methods

### 2.1. Study Design and Area

An institutional-based cross-sectional study was carried out to determine the prevalence of *N. gonorrhoeae* and its antimicrobial susceptibility patterns at private clinics found in Jimma Town, Ethiopia.

### 2.2. Sample Size Assumption and Sampling Technique

The sample size was determined with single population proportion formula assuming the prevalence of gonococcus infection 5.1%, as it was reported by a study done in Hawassa [12], 95% confidence interval (CI) and margin of error half of *P* (0.0255). In addition, considering 10% nonresponse rate, the total sample size was 315. From thirteen private clinics found in the town, three clinics were selected using simple random sampling technique and the patients were enrolled consecutively during the study period.

### 2.3. Data Collection

The data were collected by trained health professional via face-to-face interview technique using a structured questionnaire to collect sociodemographic and other data from study participants. The questionnaire was adopted after reviewing different studies done before.

### 2.4. Isolation and Identification of *Neisseria gonorrhoeae*

After properly collected, urogenital specimens were inoculated on Modified Thayer Martin medium and were incubated in candle jar at 35–37°C for 72 hours and then the plate was inspected for growth. If there is growth, further biochemical test was done to confirm *N. gonorrhoeae*. The organism is oxidase positive and ferments glucose but not maltose, sucrose, or lactose.

### 2.5. Antimicrobial Susceptibility Testing

Antimicrobial susceptibility testing of isolated *N. gonorrhoeae* was performed by using the Kirby–Bauer disk diffusion test, according to CLSI [13]. From the pure culture, 3–5 colonies of bacteria were transferred to a tube with sterile normal saline to prepare a suspension which is comparable with 0.5 McFarland standards. A sterile swab was used to distribute the bacteria evenly over the entire surface of chocolate agar with 1% selective supplement. The susceptibility patterns of the isolates were tested against the following antimicrobial agents: penicillin (P 10 IU), tetracycline (TE 30 *μ*g), ciprofloxacin (CIP 5 *μ*g), ceftriaxone (CRO 30 *μ*g), and spectinomycin (SPT 100 *μ*g); all are from Oxoid. The standard reference strain of *N. gonorrhoeae* ATCC 49226 was used as recommended by the Clinical and Laboratory Standards Institute (CLSI) to control the overall quality [13].

### 2.6. Data Management and Statistical Analysis

The collected data were entered in Epi Data version 3.1 and transferred to a statistical package for Social Sciences (SPSS, version 25) for analysis. Bivariate analysis was employed to identify factors associated with gonococcal infection. Multivariate analysis was performed for those factors that showed a *P* value ≤0.25 significant association in bivariate analysis and to investigate independent predictors by controlling for possible confounders. *P* value ≤0.05 was considered statistically significant.

## 3. Results

### 3.1. Sociodemographic Characteristics of the Study Population

A total of 315 patients were included in the study. Of those majority, 301 (95.6%) were females. The age ranging from 20 to 24 years was 144 (45.8%) with a mean age of 25.5 ± 7.5 years. Two hundred forty (76.2%) of participants were from urban and the rest, 75 (23.8%), were from rural. Regarding their marital status, the majority, 187 (59.4%), were single and more than one-fifth of the participants (72 (22.9%)) were students (Table 1).

### 3.2. Prevalence of Gonococcal Infection

Among 315 participants, 31 (9.8%) were culturally confirmed to have a gonococcal infection. Of these, 30 (96.77%) were females. From 31 total positive cases, 20 to 24 years groups were more affected which is 18 (58.1%). In our study, out of the total of 31 positive cases, 18 (58.1%) were from urban and 13 (41.9%) were from a rural setting. There is a statistically significant association between place of residence and gonococcal infection (*P* ≤ 0.007) and the odds of having gonorrhea infection to people living in a rural area were three times higher than their urban counterparts (OR (95% CI) = 3.07 (1.36–6.95)). Three (33.3%) and 9 (12.5%) of the gonococcal positive cases were seen in those divorced and students, respectively (Table 1). The prevalence of gonococcal infection with respect to sexual behaviors is high among participants who have multiple sexual partners, 23 (37.7%) (Table 2).

Concerning use of condom, the risk of gonococcal infection is higher among condom users; it might be due to improper use during sexual intercourse. In this study, the prevalence of gonococcal infection among people who have no basic information about STI is higher than the study participants who have awareness about STI (18 (16.07%)). In our study, the prevalence of gonococcal infection concerning alcohol use and “Khat” chewing is 3 (7.5%) and 2 (6.67%), respectively (Table 2).

### 3.3. Antimicrobial Resistance Patterns of the Isolates

**Figure 1:**
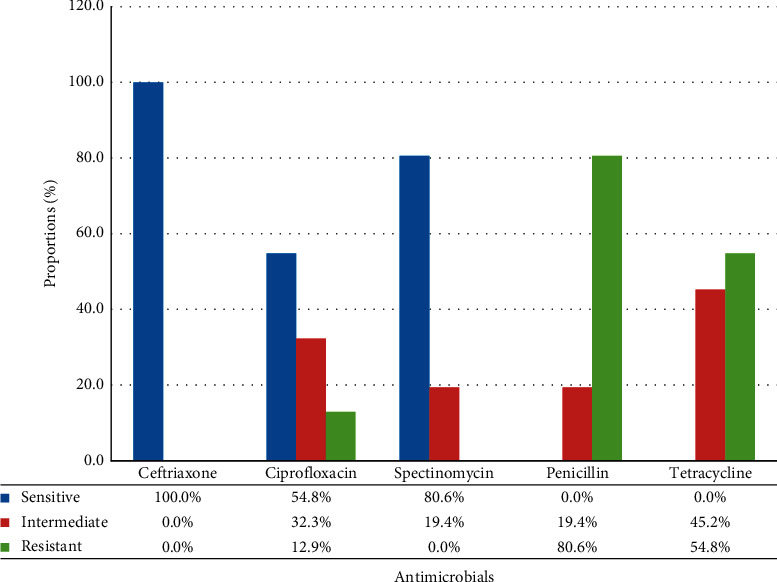
Antimicrobial susceptibility patterns of *N. gonorrhoeae* isolated from patients suspected of gonococcal infection.

In our study, the susceptibility patterns of isolated bacteria (*n* = 31) were done against 5 antimicrobial agents by the agar disc diffusion technique. The susceptibility patterns of gonococcal isolates range from 100% (ceftriaxone) to 0% (penicillin and tetracycline). The lowest susceptibility was observed for penicillin and tetracycline. No resistance was found to ceftriaxone. However, a low level of susceptibility to quinolones (ciprofloxacin 54.8%) was observed, which was recommended in the national protocol as first-line drugs for gonorrhea treatment. There was decreased susceptibility to spectinomycin as well (80.6%) (Figure 1).

## 4. Discussion

According to the World Health Organization (WHO) and Pan America Health Organization (PAHO) report, *N. gonorrhoeae* infection was the second most common bacterial STI and results in substantial morbidity and economic cost worldwide [14] and the highest prevalent sexually transmitted disease (STD) in low-income and middle-income countries [15]. In Ethiopia, several studies were done on the prevalence and antimicrobial susceptibility patterns of *N. gonorrhoeae* but comparing the result of these studies is not simple due to the difference in methodology of the studies, sample sizes, and study areas.

The prevalence of gonococcal infection in this study is lower than the finding reported in Nigeria 28.1% [16]. On the contrary, this finding is higher compared to other findings reported from Jordan 2.2% [17], Ghana 0.9% [18], and Kenya 0.4% [19]. The prevalence of this study is comparable with a study reported in Bahirdar (8.2%) [20], lower than the study in Gondar (12.07) [21] and higher than a study reported from Hawassa (5.1%) [12].

Regarding sex and age, in the present study, the prevalence of gonococcal infection was higher in females 30 (96.8%), and, in the age group 20–24 years, 18 (12.5%). This finding has comparable proportions with the finding reported from Jimma where 68.3% females and 31.7% males were reported as infected [22]. This might be due to these age groups being sexually active age groups which are at high risk of gonococcal infection. Patients who came from rural areas have increased risk of developing gonococcal infections (OR (95% CI) = 3.07 (1.36–6.95), *P* ≤ 0.007) and its prevalence rate is 13 (17.30%). This study is similar to the study done in Hawassa where the prevalence rate was 2.8% [12]. The prevalence of gonococcal infection concerning marital status was highly reported in divorce, which accounted for about 3 (33.3%) and it was significantly associated with gonococcal infections (OR (95% CI) = 6.9 (1.5–32.5); *P* ≤ 0.015). Our study is comparable with the study conducted in Thailand, 29.3% [23].

The rate of gonococcal infection was higher in participants who had multiple sexual partners (23 (37.7%)), which is similar to the study done in Jimma [22]. Our results also showed that the majority of the respondents who did use condom during sexual intercourse were highly infected (12 (13.64%)) in contrast to the study conducted in Jimma [22]. According to this study, the prevalence of gonococcal infection is higher among patients who have no awareness about STI (18 (16.07%)). This study is in line with the study conducted in Jimma [22].

The knowledge of antimicrobial susceptibility is a prerequisite for the proper treatment and control of gonococcal infection. A regional program for monitoring gonococcal antimicrobial susceptibility has been established in developed countries [24]. However, in developing countries, the burden of disease is high and the drug resistance pattern is extensively increasing. Although it is recommended in the national protocol as first-line antibiotics for gonorrhea treatment, in our finding, only 54.8% of ciprofloxacin susceptibility was observed. This is comparable with other studies, 55% in Ethiopia [25], 61% in the USA [26], and 51% in Australia [27]. In our study, spectinomycin was found to be effective against 80.6% isolates. This is similar to the study conducted in Hawassa (80.6%) [12] and Bahirdar (76.4%) [20]. This might be because of the intensive use of the antimicrobial agent, easy availability, and irrational use of this drug without laboratory diagnosis and it will be a real concern about gonorrhea because it will soon become untreatable with these antibiotics [28].

In this finding, a high level of resistance to penicillin (80.6%) and tetracycline (54.8%) was observed which is comparable with other studies in the USA [29], Australia [30], and Romania [9]. It might be due to the emergence of penicillin-resistant beta-lactamase-producing strains. A high prevalence of plasmid-mediated high-level or chromosomally mediated resistance to penicillin or tetracycline has been reported in Southeast Asia [31] and South Africa [27]. Also, it is known that gonorrhea and drug resistance vary greatly among countries and regions, because of the treatment algorithm and the way the case diagnosed and treated varies in every region. According to the syndromic case management principle set by the Ministry of Health in Ethiopia [32], the drugs (ciprofloxacin, tetracycline, and spectinomycin) have been prescribed for patients suspected of gonococcal infections.

## 5. Conclusion

In the present study, the prevalence of gonococcal infection is high, and rural residents are three times more likely to be infected with gonococcus than their urban counterparts. Having multiple sexual partners and lack of information about sexually transmitted infections (STIs) were the important risk factors for *N. gonorrhoeae* infection. In our finding, high level of resistance to penicillin and tetracycline was observed. Therefore, proper laboratory diagnosis and antimicrobial susceptibility testing are highly recommended for proper management of patients infected with gonococcus.

## Figures and Tables

**Table 1:** Description of the demographic data of patients investigated for gonococcal infection at Jimma Town Private Clinics, Jimma, Ethiopia.

Variable	Gonococcal infection		Total	COR		AOR	
	Yes (%)	No (%)		95% CI	*P* value	95% CI	*P* value
*Sex*							
Male	1 (3.2)	13 (4.6)	14 (4.4)	1.0			
Female	30 (96.8)	271 (95.4)	301 (95.6)	0.69 (0.088–5.499)	0.730		

*Age*							
15–19	2 (5.9)	32 (94.0)	34 (10.9)	1.4 (0.5–3.8)	0.502	1.3 (0.3–6.2)	0.707
20–24	18 (12.5)	126 (87.5)	144 (45.8)	2.1 (0.6–6.8)	0.239	1.8 (0.4–9.0)	0.468
25–29	7 (8.3)	77 (91.7)	84 (26.8)	1.3 (1.2–3.2)	0.840	0.9 (0.1–10.3)	0.936
30–34	1 (11.1)	8 (88.9)	9 (2.9)	2.1 (0.9–1.3)	0.241	0.8 (0.5–4.9)	0.671
≥35	3 (6.8)	41 (93.2)	44 (13.6)	1.0		1.0	

*Residence*							
Urban	18 (7.5)	222 (92.5)	240 (76.2)	1.0	0.150	1.0	
Rural	13 (17.3)	62 (82.7)	75 (23.8)	0.4 (0.2–0.8)		3.0 (1.3–6.9)	0.007

*Marital status*							
Married	10 (8.4)	109 (90.3)	119 (37.8)	1.0	0.720	1.0	0.010
Single	18 (9.6)	169 (91.6)	187 (59.4)	1.1 (0.5–2.6)	0.039	7.5 (1.6–34.5)	0.015
Divorced	3 (33.3)	6 (66.67)	9 (2.8)	0.2 (0.1–0.9)		6.9 (1.5–32.5)	

*Income*							
≤500	17 (9.6)	161 (90.5)	178 (56.5)	0.9 (0.4–2.18)	0.966		
501–999	3 (15)	17 (85)	20 (6.3)	0.6 (0.2–2.3)			
≥1000	11 (9.4)	106 (90.6)	117 (37.2)	1.0	0.449		

*Edu. Level*							
Illiterate	0	19 (100)	19 (6.0)	1.1 (0.4–2.8)	0.833		
Grade 1–8	9 (10.9)	73 (89.0)	82 (26.0)	0.6 (0.1–2.1)	0.407		
Grade 9–12+	22 (10.3)	192 (89.7)	214 (68.0)	1.0			

**Table 2:** Sexual behavior and awareness of patients towards gonococcal infections at Jimma Town Private Clinics, Jimma, Ethiopia.

Sexual behavior		Gonococcal infection		Total (%)	COR		AOR	
		Yes (%)	No (%)		95% CI	*P* value	95% CI	*P* value
>1 partner	Yes	23 (37.7)	38 (62.3)	61 (19.4)	0.66 (0.28–1.56)	0.342		
	No	8 (3.1)	246 (96.9)	254 (80.6	1.0			

Condom use	Yes	12 (13.6)	76 (86.4)	88 (27.9)	1.0	0.160	1.0	0.163
	No	19 (8.4)	208 (91.6)	227 (72.1)	0.0 (-.0–0.1)		0.6 (0.2–0.3)	

Information about STIs	Yes	13 (6.4)	190 (93.6)	203 (63.8)	1.0			
	No	18 (16.1)	94 (83.9)	112 (36.2)	0.8 (0.2–2.9)	0.820		

Alcohol use	Yes	3 (7.5)	37 (92.5)	40 (12.7)	1.3 (0.5–3.3)	0.568		
	No	28 (10.2)	247 (89.8)	275 (87.3)	1.0			

Khat chewing	Yes	2 (6.7)	28 (93.3)	30 (9.5)	0.9 (0.3–3.4)	0.976		
	No	29 (10.2)	256 (89.8)	285 (90.5)	1.0			

STI = sexually transmitted infection.

## Data Availability

The data supporting the finding of this study are included within the article.

## Additional Points

*Limitations*. The limitation of the current study was the study design; in a cross-sectional study, it is difficult to infer the cause-effect relationships for risk factors. Other limitations that are most investigated information such as Khat chewing, alcohol use, and STIs was culturally not acknowledged and liable to social desirability bias.
